# MASLD-Related HCC—Update on Pathogenesis and Current Treatment Options

**DOI:** 10.3390/jpm14040370

**Published:** 2024-03-30

**Authors:** Catherine Leyh, Jason D. Coombes, Hartmut H. Schmidt, Ali Canbay, Paul P. Manka, Jan Best

**Affiliations:** 1 Department of Gastroenterology, Hepatology and Infectious Diseases, University Hospital Düsseldorf, Medical Faculty of Heinrich Heine University Düsseldorf, 40225 Düsseldorf, Germany; 2 Internal Medicine, Division of Gastroenterology and Hepatology, Saint Louis University School of Medicine, Saint Louis, MO 63104, USA; jason.coombes@health.slu.edu; 3 Department of Gastroenterology, Hepatology and Transplant Medicine, Medical Faculty, University of Duisburg-Essen, 45147 Essen, Germany; 4 Department of Internal Medicine, University Hospital Knappschaftskrankenhaus Bochum, Ruhr University Bochum, 44801 Bochum, Germany

**Keywords:** hepatocellular carcinoma, HCC, MASLD, MASH, NAFLD, NASH, biomarker, intestinal microbiota

## Abstract

Hepatocellular carcinoma (HCC) is a common complication of chronic liver diseases and remains a relevant cause of cancer-related mortality worldwide. The global prevalence of metabolic dysfunction-associated steatotic liver disease (MASLD) as a risk factor for hepatocarcinogenesis is on the rise. Early detection of HCC has been crucial in improving the survival outcomes of patients with metabolic dysfunction-associated steatohepatitis (MASH), even in the absence of cirrhosis. Understanding how hepatocarcinogenesis develops in MASH is increasingly becoming a current research focus. Additive risk factors such as type 2 diabetes mellitus (T2DM), genetic polymorphisms, and intestinal microbiota may have specific impacts. Pathophysiological and epidemiological associations between MASH and HCC will be discussed in this review. We will additionally review the available tumor therapies concerning their efficacy in MASH-associated HCC treatment.

## 1. Introduction

Hepatocellular carcinoma (HCC) is one of the leading causes of cancer-related deaths worldwide [1]. Hepatocarcinogenesis is predisposed by liver cirrhosis of any etiology. However, chronic viral hepatitis B and metabolic dysfunction-associated steatohepatitis (MASH), formerly known as non-alcoholic steatohepatitis (NASH) [2], already significantly increase the risk of developing HCC, even in the absence of cirrhosis [3,4,5]. 

The increasing prevalence of metabolic dysfunction-associated steatotic liver disease (MASLD), previously known as non-alcoholic fatty liver disease (NAFLD) [2], and its progression to MASH raise the incidence of progressive liver fibrosis, cirrhosis, and HCC. Current data support the notion that MASH is the leading cause of the predicted increase in HCC incidence in the next decades [6].

Causal therapeutic approaches to HCC-predisposing MASH are taking place in the context of clinical trials, which is why weight normalization, exercise, and optimization of concomitant diseases such as diabetes mellitus are still the current focus. Understanding how hepatocarcinogenesis develops in MASH is increasingly becoming a key research focus. Here, the roles of additive risks such as type 2 diabetes mellitus (T2DM), genetic polymorphisms, and the intestinal microbiome are of particular interest. 

The importance of HCC surveillance in the MASH population is controversial. On the one hand, there is a lack of international consensus regarding the risk population to be defined. On the other hand, it has not been clarified whether standard screening methods using sonography in patients with MASH have sufficient sensitivity in early HCC detection [7,8].

In recent years, there has finally been a breakthrough in the systemic therapy of HCC, so several therapeutic options are now available in addition to current locoregional procedures. Since MASH is associated with a multitude of comorbidities, it remains challenging to extract the individual impact of MASH on the efficacy of available treatment options. This is particularly important when comparing MASH-related HCC with the HCC of other etiologies. 

## 2. Epidemiology of HCC in MASH

Primary liver carcinomas represent the sixth most common cancer worldwide and the third leading cause of cancer-related death [1]. Approximately 75–90% of these tumors are classified as primary HCC, and only 10–15% of cases are classified as cholangiocarcinoma (CCA) [9]. The annual HCC incidence is almost identical to its annual mortality, illustrating the high mortality of this disease [10,11]. 

In the Western world, chronic viral hepatitis C is currently shown to be responsible for the majority of all new HCC cases. However, a gradual decline is expected in the coming years due to the success of direct antiviral therapies (DAAs) [12]. Alcohol-induced liver disease also accounts for 10–20% of relevant HCC predisposing factors, and chronic viral hepatitis B accounts for 10–15% of cases. In older epidemiological analyses, a large proportion of patients with MASH were not recorded separately and were, thus, incorrectly assigned to the cohort of “cryptogenic” cirrhosis. This can partly explain divergent data on the frequency of cryptogenic liver disease (15–50%) as an HCC risk factor [13].

According to the most recent epidemiological studies, hepatic steatosis is the leading cause of the increasing incidence of HCC in Western industrialized nations. An analysis of the US Liver Transplant Registry between 2002 and 2017 found that the prevalence of patients with MASLD awaiting liver transplantation increased by 16% over the past 15 years, from 6% in 2002 to 22% in 2017, with MASH emerging as the fastest growing cause. The proportion of MASH as a cause of HCC increased 8.5-fold during the study period, from 2% in 2002 to 18% in 2017 [14]. It should be noted here that a high number of HCC cases arise against a background of MASH in the absence of cirrhosis [3]. This population has not been subject to international consensus screening for early HCC detection. 

Another analysis of patients transplanted for end-stage liver disease between January 2002 and December 2016 using the European Liver Transplant Registry database showed a greater proportion of patients transplanted for MASH-associated HCC (39.1%) than non-MASH patients (28.9%, *p* < 0.001) [15].

A US cohort study of over 500,000 participants, published in 2018, showed a cumulative 5- (or 10)-year HCC risk of 0.8 (or 1.7) per 1000 patients in the MASLD cohort. Compared with healthy controls, patients with MASLD had an 8.6-fold increase in HCC risk. Patients with MASLD-associated cirrhosis showed a further marked increase in HCC risk, with an annual incidence of 0.8–2.3% per year. Approximately 20% of all hepatic steatosis-associated HCC cases occurred without predisposing cirrhosis [11].

A large German monocentric study with 1119 HCC patients, in 2015, demonstrated epidemiological differences between patients with MASH-related HCC and those with other HCC-predisposing liver diseases. For instance, patients with MASH-related HCC were older at initial diagnosis than others (68 vs. 65 years). The MASH-HCC cohort showed a higher prevalence of obesity (31.1% vs. 14.7%) and T2DM (66.7% vs. 37.85%), with higher body mass index (BMI) correlating with worse overall survival. In MASH patients, there was a trend toward multifocality (80% vs. 70%) with larger lesions overall (6.0 cm vs. 4.8 cm). In addition, there was a tendency toward an increased extrahepatic metastasis rate at the initial diagnosis. Interestingly, liver function was preserved to a greater extent than in other etiologies of HCC patients. Therefore, it can be postulated that the potential diagnosis of MASH-associated cirrhosis and its complications occurred later. As a result, HCC could only be detected at more advanced stages [16]. Consequently, optimization of current HCC screening algorithms and a more precise definition of the population to be screened seem all the more urgent.

Projections using dynamic Markov modeling for accounting for obesity and diabetes trends claim that the annual incidence of MASLD-related HCC in the USA would increase by 137% from 5160 cases in 2015 to 12,240 cases in 2030 [17]. 

## 3. Pathogenesis of HCC in MASH

**Figure 1:**
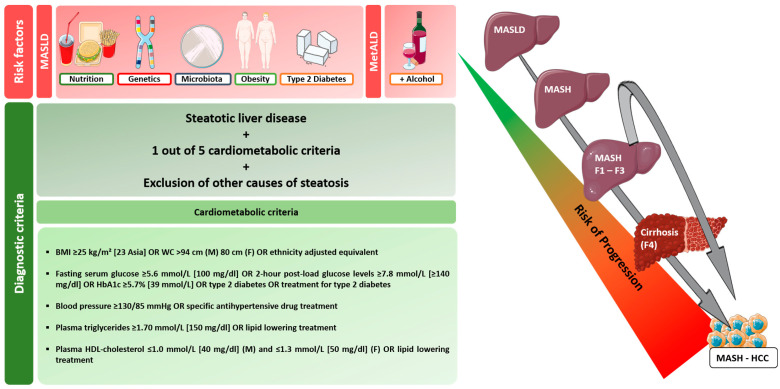
Diagnostic criteria and risk factors for MASLD and progression of MASLD to MASH-HCC [19]. In the progression from MASLD to MASH, MASH fibrosis, and finally cirrhosis, a variety of factors (type 2 diabetes mellitus, obesity, microbiome, genetic and epigenetic factors, lifestyle) have a similar effect on hepatocarcinogenesis. HCC risk appears to correlate with the extent of fibrosis, but MASH-associated HCC may develop even without cirrhosis. The time to which the above factors, particularly type 2 diabetes, have direct carcinogenic potential has not been conclusively determined. Abbreviations: MetALD: metabolic dysfunction and alcohol-associated steatotic liver disease, BMI: body mass index; WC: waist circumference.

Chronic hepatic inflammation in the setting of MASH represents a potential trigger of hepatocarcinogenesis even in the absence of predisposing cirrhosis. It is a complex, multifactorial process involving various risk factors (genomic instability, obesity, diabetes mellitus, and others) (Figure 1). Metabolic alterations (lipid and glucose metabolism) contribute to hepatic steatosis. However, additional factors such as genetic variants, oxidative/endoplasmic reticulum stress, mitochondrial dysfunction, altered immune response, and microbiome conditions fuel disease progression (hepatic inflammation, fibrogenesis, and carcinogenesis) [18]. For conciseness, we focus on the influence of genetics and polymorphisms. In addition, the microbiome’s impact on the progression from MASH to HCC will be considered separately. 

### 3.1. Genetic Factors

Several gene polymorphisms have demonstrated an association between the prevalence of MASLD per se and the risk of progression to advanced MASH fibrosis [20]. The best known is the polymorphism of the PNPLA3 (patatin-like phospholipase domain containing 3) gene (variant rs738409 c.444 C>G, p.I148M) on chromosome 22. This PNPLA3 variant leads to impaired triglyceride mobilization of hepatic lipid droplets with increased hepatic lipid accumulation, but also the alteration of retinol storage in the liver, and altered retinol serum levels, especially in obese patients. Of note, the PNPLA3 polymorphism is associated with a 3-fold increased risk of HCC in its carriers, independent of other risk factors such as BMI, diabetes, and advanced fibrosis [21]. 

The TM6SF2 (transmembrane 6 superfamily member 2) gene polymorphism (variant rs58542926, c.449 C>T, p.E167K) on chromosome 19 manifests as a transport disorder of pre-VLDL particles. It correlates with the extent of steatosis and progression of fibrosis in MASH, independent of obesity, diabetes, and the PNPLA3 genotype. However, the direct role of this TM6SF2 variant in hepatocarcinogenesis is controversial; the profibrogenic effect might indirectly promote progression to HCC [22,23].

Recent data also suggest that a loss-of-function variant in the 17-beta hydroxysteroid dehydrogenase 13 gene (HSD17B13) is associated with a reduced risk of chronic liver disease and of progression from steatosis to steatohepatitis and, thus, may represent another factor in HCC development [24,25,26]. 

In addition, individual studies of different mutations have demonstrated an unfavorable influence on the course of MASH. Mutations of the hereditary hemochromatosis protein-encoding gene (HFE) on chromosome 6 are associated with a complicated course of MASH, potentially favoring the development of HCC [27]. 

In conclusion, it is important to note that both hepatocarcinogenic single mutations and the increased genetic instability in patients with MASH, compared to those with MASLD, favor the development of HCC. Other mechanisms promoting the progression of MASH to HCC include epigenetic alterations causing aberrant DNA methylation and the expression of diverse microRNAs (miR-21, miR-23, miR-29, miR-93, miR-106, miR-155, miR-221, miR-222, and miR-519). Mechanisms and pathways involved are the major tumor-associated signaling cascades (transforming growth factor (TGF-), Wingless and INT-1 (Wnt)/catenin, mitogen-activated protein kinase (MAPK), Hedgehog, NF-κB, phosphatidylinositol 3-kinase (PI3K)/AKT/Mechanical (mammalian) target of rapamycin (mTOR)) [28], and CD44 [29].

### 3.2. Intestinal Microbiome

The gut microbiome is considered a key modulator of metabolism. It not only facilitates the extraction of nutrients and energy from food but is also essential for producing numerous metabolites, including bile acids, regulating various metabolic pathways. The gut microbiome outnumbers the human genome many times over. It plays a vital role in metabolism, immune system formation, health maintenance, tolerance development, and the prevention of colonization by pathogens. Its alteration, called dysbiosis, has been described for different intestinal metabolic and inflammatory diseases, including MASLD/MASH, alcoholic liver disease, cirrhosis, and complications [30,31,32,33]. 

Shifts of specific bacterial strains affect the production of bacterial-derived metabolic active components, including bile acids, ethanol, cytokines, short-chain fatty acids, or other inflammatory metabolites. Those may affect the host and possibly promote cancer-related risk factors or diseases [34]. In rodent studies, fecal microbiota transplantation increased the abundance of beneficial bacterial groups and alleviated the progression of MASH development [35]. 

Modulating the gut microbiota, e.g., with antibiotic treatment, may reduce the risk of hepatic carcinogenesis [36,37]. After antibiotic treatment, mice fed a high-fat diet showed reduced toxic secondary bile acids [38]. In MASLD and especially MASH, nutrition, metabolic disturbances, and related comorbidities such as diabetes may influence gut microbiota composition. Changes in the abundances of different bacterial groups have been described within other patient groups. The metabolism of specific bacterial groups affects the mucosal barrier, hepatic inflammation, fibrogenesis, and tumorigenesis [39]. The gut microbiota impacts energy balance, altering the uptake of calories derived from food and alcohol [40]. Emerging data indicate that specific characteristic changes in the gut microbiome are associated with MASLD and even cirrhosis, which is the primary driver of HCC development [31,41,42]. In MASLD-related HCC and viral hepatitis-related HCC (hepatitis B), specific modification of gut microbiota may represent a potential therapeutic option for HCC treatment [43]. A study comparing MASH and MASH-HCC patients with or without cirrhosis showed that changes in bacterial groups regulating bile acid metabolism affected hepatic fibrogenesis and liver injury. The changes in the bile acid pool were associated with an increased abundance of several bacterial strains, particularly Lactobacilli and Bacteroides, which were related to altered liver injury and liver stiffness [44]. In patients with MASH, the abundance of bile salt hydrolase-expressing bacteria is shifted, resulting in increased bile acid levels and the altered composition of the bile acid pool, which tends to increase the amount of secondary conjugated bile acids [45]. Alterations in bile acid composition may be associated with advanced fibrosis in MASH-HCC, suggesting an essential role in fibrosis-related tumorigenesis [46,47]. 

## 4. HCC Surveillance in MASLD/MASH

It is well established that patients participating in surveillance programs are diagnosed with HCC at less advanced stages, resulting in a survival advantage. Successful screening, however, requires reliable screening methods, on the one hand, and a definition of the population at risk, on the other hand.

In the context of a steady worldwide increase in HCC incidence on the background of MASH, there is a compelling need for a clear recommendation regarding HCC surveillance for this patient population. While the risk of HCC in MASH cirrhosis is sufficiently high to warrant surveillance, there is still no clear consensus among international guidelines for patients with MASH without cirrhosis. The German HCC S3 guideline provides sonographic screening with or without additional AFP determination every six months in MASH patients, even in non-cirrhotic livers. The current AASLD (American Association for the Study of Liver Diseases) HCC guidelines do not recommend surveillance in MASLD alone; only in MASH cirrhosis should sonography (±AFP determination) be offered every six months [48]. The 2018 EASL (European Association for the Study of the Liver) HCC guidelines also do not recommend general HCC surveillance in MASH; however, surveillance may be considered across etiologies in patients with advanced fibrosis (F3) without cirrhosis based on the individual risk profile. The EASL guidelines only recommend sonographic controls every six months without additional determination of AFP (European Association for the Study of the Liver). 

As early as 2011, Ertle et al., in a retrospective epidemiological HCC monocenter study, described that 42% of patients in the MASLD/MASH-HCC group did not have cirrhosis at the time of HCC diagnosis [3]. Later publications showed a slightly lower proportion of non-cirrhotic MASH-HCC patients ranging from 23% to 37% [49,50]. 

The sensitivity of ultrasound-based surveillance in MASH patients is limited by investigator dependence, frequent concomitant obesity, and by MASH itself. A recent US meta-analysis examined the sensitivity of sonography in HCC detection, with or without concurrent AFP determination, in a cohort of 13,367 high-risk HCC patients of any etiology. Ultrasound alone showed a sensitivity of only 45%, which significantly increased to 63% with the addition of AFP. Thus, whether sonography alone can reduce HCC-associated mortality remains inconclusive and urgently requires data from prospective studies [51].

To address the unsatisfactory performance of sonography-based HCC surveillance, the GALAD score was developed, which includes age, sex, and the biomarkers AFP, AFP isoform L3 (AFP-L3), and Des-γ-Carboxy-Prothrombin (DCP), also known as protein induced by vitamin K absence or antagonist-II (PIVKA-II). In a recent Japanese–German multicenter study, it was shown in MASH patient collectives that the GALAD score can detect HCC with high sensitivity while comprising reasonable specificity in these patients in the presence and absence of cirrhosis. Even early-stage HCC could be detected with high reliability [52]. However, a limitation of this multicenter study is the lack of a direct comparison with an ultrasound-based surveillance strategy in the populations studied. A future prospective study should, therefore, investigate the performance of ultrasound alone and in combination with the GALAD score in NASH patients. A retrospective American multicenter study shows that this approach may be promising, with the combination of GALAD and ultrasound achieving an AUC of 0.98 (US alone AUC 0.82 vs. GALAD alone AUC 0.95) [53].

Recent data show that hepatocarcinogenesis is accompanied by epigenetic changes and mutations in various genes. These molecular changes, which have so far been described using tissue biopsies and cell models, can also be detected in circulating fragmented DNA (cfDNA) in the blood. As with other tumor entities, the molecular cfDNA can be used to analyze the molecular changes [54]. In this context, it would be all the more obvious to establish the use of liquid biopsy (LB) in HCC on a larger scale in order to be able to do justice to the still insufficient early diagnosis. The use of LB appears to be particularly useful in HCC, as the arterial hyperperfusion of tumor tissue means that there is a particularly high probability of circulating tumor cells (CTCs) and cfDNA being distributed in the peripheral blood.

## 5. Therapeutical Challenges in Patients with MASH-Related HCC

In unifocal HCC (Barcelona Clinic Liver Cancer (BCLC) stage 0/A), therapy of smaller lesions with curative intent is possible by radiofrequency ablation (RFA) or microwave ablation (MWA). For larger lesions, liver resection is indicated, assuming there is preserved liver synthesis function in the absence of portal hypertension. A large US monocenter study demonstrated that patients with HCC and MASH had better liver function at the time of resection and showed a lower rate of cirrhosis than patients with hepatitis C or alcohol-related liver disease. As a result, a more significant proportion of patients could be resected in MASH-associated HCC compared with the other etiologies. In addition, improved overall survival (OS) has been detected compared with the HCV/alcohol cohort [55].

For functionally irresectable HCC with a single lesion <5 cm or a maximum of 3 lesions <3 cm (Milan criteria), evaluation for liver transplantation (LT) is recommended. It should be emphasized at this point that, as mentioned at the outset, MASH is primarily responsible for the sharp increase in HCC-related transplant listings in the United States. 

The resulting increase in demand for donor organs requires more optimized allocation of organs. However, current data suggest that the Milan criteria, which are still considered the standard, could deny many potential organ recipients suffering from MASLD-associated HCC from receiving a donor organ due to the more advanced tumor stages at initial diagnosis. In recent years, several different approaches have been taken to extend these current criteria. Among the best known are the UCSF criteria, defined as a single tumor that is less than or equal to 6.5 cm, or up to 3 lesions with the largest lesion less than or equal to 4.5 cm, with a total tumor diameter no greater than 8 cm. Here, patients transplanted within these criteria had a 5-year survival rate of 75.2% [56]. In contrast, the Up-to-Seven criteria propose that for HCC, with 7 as the total of the size of the largest lesion in cm and the number of lesions, and without vascular invasion, could have survival outcomes as good as those within the Milan criteria. In the patient cohort without microvascular invasion but within the Up-to-Seven criteria, 5-year overall survival was 71.2% [57]. Later, Mazzaferro et al. developed the Metroticket 2.0 model, incorporating the size of the largest HCC lesion, the total number of nodules, and the AFP level in 2018. According to this model, the overall 5-year survival rate reached 79.7%, and tumor recurrence could be more accurately estimated than with the Milan, UCSF, or Up-to-7 criteria [58]. 

The additional use of biomarkers in decision algorithms for LT is a matter of debate. However, there is a correlation between microvascular invasion (MVI) [59], histological differentiation, and the risk of post-LT HCC recurrence, as reported in several publications. AFP and DCP levels are reliable surrogate markers of the biological behavior of HCC due to their strong correlation with MVI and degree of differentiation [60] and micro-intrahepatic metastasis. The inclusion of these markers in the development of criteria has become widely accepted in the last decade.

Even at the listing stage, MASH presents unique features compared to other etiologies. It is well known that metabolic syndrome (especially T2DM and obesity), related coronary artery disease, and chronic renal failure are predictors of worse postoperative and long-term outcomes. On the LT waiting list, patients with MASH frequently exhibit numerous metabolically related risk factors simultaneously. This results in several potential pitfalls for LT-listed patients with MASH: an overall older patient age at the time of listing for liver transplantation, comorbidities, and a lower model for end-stage liver disease (MELD) score than other etiologies. In the context of obesity and transplantation, the results of different meta-analyses are wildly divergent. Some studies report worse post-LT survival in patients with high BMI, whereas other papers report similar long-term outcomes as in normal-weight patients. The postoperative complication rate is higher in obese patients. Whether combining bariatric surgery with LT may improve overall survival needs to be investigated using more extensive randomized trials. However, T2DM should be emphasized as a predictor of poor graft and long-term patient survival. In the future, the integrated weighting of MASH-associated comorbidities in selecting MASH-HCC patients for potential LT listing will be a significant challenge [61].

### 5.1. Treatment of Intermediate-Stage HCC

For patients presenting non-diffuse, multifocal tumor nodules without extrahepatic tumor manifestations or macrovascular invasion and compensated liver function (BCLC stage B), a transarterial chemoembolization (TACE) treatment is recommended, according to the EASL guidelines [62]. A survival benefit from TACE was first demonstrated in 2002 in a randomized controlled trial comparing TACE to symptomatic treatment [63]. Since then, the technique has undergone several improvements, e.g., super-selective chemoembolization of the target volume can facilitate optimal sparing of healthy liver tissue surrounding the tumor and, thus, help preserve liver function. As a different concept for locoregional therapy, transarterial radioembolization (TARE) is an alternative treatment option for patients with locally advanced HCC who are not eligible for TACE [62]. To the best of our knowledge, little evidence exists regarding MASLD’s influence on the efficacy and safety of locoregional therapies. 

In a retrospective study comparing the outcome of TACE in cirrhotic patients with MASH-related HCC to patients with HCV- or alcohol-related HCC, no significant differences in OS, response rate, or time to progression (TTP) could be identified. Apart from adverse events, no significant differences between the groups were also observable [64]. Concerning MASLD-related comorbidities, another retrospective study could identify obesity (BMI ≥ 25 kg/m^2^) as a risk factor for worse tumor response after TACE [65]. Furthermore, T2DM could be identified as an independent predictor for worse OS in patients with non-viral HCC undergoing TACE [66]. The negative influence of T2DM could be demonstrated in a further retrospective study. Patients suffering from liver cirrhosis and concomitant T2DM had a worse long-term prognosis after TACE [67]. Interestingly, the drug Metformin, frequently prescribed for T2DM, seems to have beneficial effects on the outcomes after TACE in patients with T2DM and early-stage HCC [68]. Regarding TARE, a retrospective study conducted by Schotten and colleagues found MASLD-related comorbidities not to influence the critical outcomes of TARE [69]. 

### 5.2. The Treatment Landscape for Advanced-Stage HCC

Over the past few years, the landscape of systemic therapy options for patients with advanced-stage HCC and compensated liver function has significantly expanded. In 2008, for the first time, two independently conducted studies (SHARP trial and Asia-Pacific trial) demonstrated a survival benefit for HCC patients treated with systemic therapy, leading to the approval of the tyrosine kinase inhibitor (TKI) sorafenib [70,71]. Based on the results of the REFLECT trial, lenvatinib was approved in 2018 as a further first-line treatment option. Although lenvatinib did not achieve superior OS (OS lenvatinib: 13.6 months vs. OS sorafenib: 12.3 months, hazard ratio (HR) 0.92, 95% confidence interval (CI) 0.79–1.06), progression-free survival (PFS), time to progression (TTP), as well as the objective response rate (ORR) significantly improved in the lenvatinib group compared to sorafenib [72]. Further, two more TKIs, regorafenib and cabozantinib, have been approved as second (or third) line therapies after pretreatment with sorafenib [73,74]. With the introduction of ramucirumab, therapy options in second-line treatment further expanded to include a monoclonal antibody directed against vascular endothelial growth factor receptor type 2 (VEGFR2). Ramucirumab, the only drug that has to be guided by a biomarker, has been approved for patients with tumor progression on sorafenib and an alpha fetoprotein (AFP) value of at least 400 ng/mL [75]. Developing the aforementioned new TKIs and monoclonal antibodies has significantly improved the OS of HCC patients eligible for systemic therapy. However, the improved OS is not consistently matched by an improved ORR. Furthermore, in HCC patients with MASH, TKI treatment approaches are potentially limited by extrahepatic manifestations of the metabolic syndrome such as obesity, diabetes, hyperlipidemia, and arterial hypertension, frequently accompanied by chronic kidney and cardiovascular disease. Here, TKIs may aggravate arterial hypertension and increase the risk of myocardial infarction, potentially necessitating treatment de-escalation or discontinuation. Therefore, in this patient cohort, alternative treatment approaches with more favorable safety profiles are required.

Regarding the critical endpoint of ORR, a clear improvement was achieved with the combination of the programmed death-ligand 1 (PD-L1) inhibitor atezolizumab and the VEGF inhibitor bevacizumab tested against sorafenib as first-line therapy (IMbrave 150 trial) [76]. In addition to a survival benefit of nearly six months (OS atezolizumab plus bevacizumab: 19.2 months vs. OS sorafenib 13.4 months), this new combination therapy induced an ORR in 30% of all participants, and 25 patients (8%) even showed a complete response. As a result, the combination of atezolizumab and bevacizumab became the new standard of care for first-line systemic treatment in 2020. Supported by the results of the above-mentioned IMbrave150 study, an immune checkpoint inhibitor (ICI)-based combination therapy is seen as having great potential in treating liver cancer. Based on the positive results of the phase III HIMALAYA trial, the dual immune checkpoint blockade consisting of the cytotoxic T-lymphocyte-associated Protein 4 (CTLA-4) antibody tremelimumab and the PD-1 inhibitor durvalumab was recently approved as an additional first-line therapy [77]. The so-called STRIDE regimen (single tremelimumab regular interval durvalumab) showed both an OS benefit (OS STRIDE 16.4 months vs. OS sorafenib 13.8 months, HR 0.78, 96% CI 0.65–0.92, *p* = 0.0035) and an improved treatment response rate (ORR STRIDE 20.1% vs. sorafenib 5.1%). In addition to combination therapy, durvalumab was also tested as a monotherapy and was found to be non-inferior to sorafenib [77].

Apart from the dual ICI blockade, the combination of an ICI with TKIs has also been tested. Recently, the results of LEAP-002, a phase III study evaluating the combination of pembrolizumab plus lenvatinib versus lenvatinib as monotherapy, were reported. Although the combination therapy did not reach the pre-specified statistical significance for OS, it achieved the longest OS among systemic treatments for advanced HCC to date (OS lenvatinib plus pembrolizumab: 21.2 months vs. OS lenvatinib: 19 months, HR 0.84, 95% CI 0.708–0.997, *p* = 0.0227) [78]. The combination of atezolizumab and cabozantinib investigated in the COSMIC 312 trial only improved PFS, but not OS [79]. In addition to their use in the first-line setting, ICIs have also been investigated in the second-line setting. For example, the CheckMate 040 phase I/II trial found that the combination of ipilimumab and nivolumab induced a durable response in a subset of patients with HCC who had previously received sorafenib treatment [34].

### 5.3. Immunotherapy and Targeted Therapies in Non-Viral HCC

Regarding the expanding therapeutic armamentarium, a new challenge arises—namely, to identify the most suitable treatment sequence for each patient as systemic treatment has been conducted in a “one-size-fits-all” fashion for many years. To what extent the etiology of the underlying chronic liver disease contributes to therapy success or failure is currently gaining increased attention. In light of the rapidly increasing incidence, patients with MASLD-associated HCC are of particular interest. A closer look into the patient cohorts of the above-mentioned phase III trials reveals that only small subgroups had non-viral HCC. Furthermore, only a few studies reported a precise number of MASLD-related etiology (Table 1) [70,71,72,73,74,75,76,80]. Interestingly, to our knowledge, only the CELESTIAL trial considered MASLD as a stratification parameter. The current knowledge regarding therapy implications for non-viral HCC is primarily based on findings from retrospective cohorts, potentially biased subgroup analyses, and meta-analyses, but no specific RCTs have been conducted assessing this issue. 

It was hypothesized that patients with non-viral HCC may benefit less from ICI-based immunotherapy. Subgroup analyses of the IMbrave150 trial indicate a clear survival benefit for viral HCC, but the HR for non-viral HCC was not statistically significant (HR 1.05, 95% CI 0.68–1.63) [76]. These findings are further supported by two meta-analyses that merged data from the three large ICI phase III trials (IMbrave 150, Checkmate 459, and Keynote 240). The analyses showed that ICI-based therapies seemed to be less effective in patients with non-viral HCC [81,82]. The possible underlying mechanisms of resistance against immunotherapy in MASLD-associated HCC are not fully understood. It seems that CD8 + PD1 + -T-cells contribute to the pathophysiology of MASLD, and interestingly, anti-PD1 therapy promotes adverse tumor necrosis factor-alpha (TNFα)-secretion by these cells in the MASLD setting [81]. However, in contrast to the aforementioned studies, newer trials reported more promising results, questioning the assumption that ICI-based therapy is not the optimal choice for patients with non-viral HCC. The recently published HIMALAYA trial demonstrated a significant survival benefit for patients with non-viral HCC receiving the STRIDE regime (HR 0.74, 95% CI 0.57–0.95) [77]. Another meta-analysis did not demonstrate a notable difference in treatment efficacy associated with the underlying HCC etiology [83]. 

Regarding TKI-based therapy, the role of the etiology of the underlying chronic liver disease is also not fully understood. A recent meta-analysis as well as a real-world study, which included patients treated with lenvatinib from German tertiary cancer centers, did not identify any differences in HCC treatment success according to the etiology of chronic liver disease [82,84]. In the SHARP trial, the survival benefit of sorafenib over the placebo was consistent for the different etiologies HBV, HCV, and alcohol. However, the effect seemed more pronounced in patients suffering from HCC due to HCV infection [85]. In a multicentric retrospective study conducted in a Japanese cohort investigating the impact of lenvatinib in accordance with the HCC etiology (MASLD/MASH group vs. Viral/Alcohol group), OS and PFS tended to be even better in the MASLD/MASH group [86]. Another retrospective study featuring a European cohort of HCC patients receiving lenvatinib treatment, conducted by Sacco and colleagues, also found that patients with non-viral HCC had longer OS compared to patients with viral-related HCC [87]. In further retrospective studies, two other working groups investigated the effect of lenvatinib compared to the combination therapy of atezolizumab and bevacizumab in patients with non-viral HCC and were able to identify a favorable effect for lenvatinib in this patient group [88,89]. In summary, etiology cannot currently serve as a selection criterion for the preferred therapy due to insufficient high-quality data from prospective studies using MASLD as a stratification criterion or even considering MASLD as a distinct subgroup. It is important to note that MASLD has also not been considered as a separate patient group in phase III studies to date and it can also be challenging to identify MASH as the sole risk factor for the development of HCC. Therefore, the combination of atezolizumab and bevacizumab remains the recommended first-line therapy for all patients. 

## 6. Future Directions

The relationship between obesity and type II diabetes mellitus in the progression of MASLD to fibrosis, cirrhosis, and HCC is well-established. Adipokines and insulin resistance are among the factors that orchestrate this progression. By effectively treating the additive risk factors of obesity and diabetes, the risk of progression to HCC can be mitigated. 

Drug therapy for morbid obesity remains a clinical challenge, which is why bariatric surgery continues to play an important role here. According to a recent large meta-analysis by Ramai and colleagues (9 studies, 18,423,546 controls vs. 1,091,204 bariatric patients), surgical treatment of obesity can significantly reduce the risk of concomitant progression from MASLD to HCC. The pooled rate per 1000 person-years was 0.05 (95% CI: 0.02–0.07) in bariatric surgery patients versus 0.34 (95% CI: 0.20–0.49) in the control group with an incidence rate ratio of 0.28 (95% CI: 0.18–0.42). Bariatric weight reduction reduces the risk of HCC in obese patients, as indicated by the data [90].

The efficacy of glucagon-like peptide-1 (GLP-1) receptor agonists such as liraglutide in the treatment of MASLD/MASH remains unclear. In an animal study, mice with streptozotocin- and high-fat diet-induced diabetes with MASH were treated with liraglutide or saline for 14 days in the control arm. The two groups were compared in terms of glycemic control: liver histology and hepato-carcinogenesis. While fasting plasma glucose was significantly lower in the liraglutide group than in the control group, fasting insulin levels were significantly higher in the test group than in the control group. Impressively, in contrast to the control group, liraglutide completely suppressed the development of HCC and also significantly attenuated steatosis and inflammation [91].

Another treatment option for the effective management of T2DM, one of the major risk factors for MASLD, is sodium-glucose co-transporter (SGLT2) inhibitors. Experimental studies in animal models have suggested that SGLT2 inhibitors may have beneficial modulatory effects on MASLD, and several studies in patients have demonstrated their beneficial effects on liver enzymes, BMI, hyperlipidemia, hyperglycemia, and insulin resistance in MASLD patients, potentially inhibiting the progression of liver damage to HCC in these patients [92]. In the future, effective control of predisposing factors for HCC will undoubtedly be an essential preventive component in reducing the increasing incidence of HCC worldwide. 

## 7. Conclusions

MASH is considered to be a significant cause of the increasing incidence of HCC worldwide. Chronic hepatic inflammation in MASH triggers hepatocarcinogenesis even without predisposing cirrhosis. It is a complex, multifactorial process involving various risk factors (genomic instability, obesity, diabetes mellitus, etc.). The heterogeneity of HCCs resulting from this multitude of MASH-associated risks makes it difficult to define a risk population to be screened clearly. In consequence, MASLD is associated with lower HCC surveillance receipt, lower early-stage cancer detection, and modestly worse OS. Furthermore, previous approaches to ultrasound-based HCC surveillance show clear limitations.

Therefore, the evaluation of alternative, e.g., biomarker-based, screening methods is mandatory. The selection of appropriate HCC treatment options for MASH patients must also consider MASH-associated and potentially therapy-limiting comorbidities, as non-cancer mortality significantly impacts OS even in curatively treated patients. Here future systemic therapy trials urgently need to address MASLD as a separate subgroup to avoid underrepresentation of this worldwide epidemic disease.

## Figures and Tables

**Table 1:** Role of HCC etiologies in selected clinical trials on systemic therapy with subsequent approval of the therapy regimen by the EMA.

Trial And Treatment Arms	Etiology *	Stratification Criteria	Primary Endpoints	Secondary Endpoints
SHARP—sorafenib vs. placebo [70]	HCV 29% HBV 19% Alcohol 26% Unknown 16% Other 9%	Geographical region ECOG PS (0 vs. 1–2) Macrovascular invasion or extrahepatic spread (presence vs. absence)	OS 10.7 vs. 7.9 (HR 0.69, 95%-CI 0.55–0.87, *p* < 0.0019) TTSP 4.1 vs. 4.9 (HR 1.08, 95% CI 0.88–1.31, *p* = 0.77)	TTRP 5.5 vs. 2.8 (HR 0.58, 95% CI 0.45–0.74, *p* < 0.001) DCR 43% vs. 32%; *p* = 0.002
Asia-Pacific—sorafenib vs. placebo [71]	HCV 70.7% HBV 10.7%	Geographical region Macrovascular invasion and/or extrahepatic spread (presence vs. absence) ECOG PS (0–2)	OS 6.5 vs. 4.2 (HR 0.68 95% CI 0.50–0.93. *p* = 0.014)	TTP 2.8 vs. 1.4 (HR 0.57, 95% CI 0.42–0.79, *p* = 0.0005) TTSP 3.5 vs. 3.4 (HR 0.90, 95% CI 0.67–1.22, *p* = 0.50) DCR 35.3% vs. 15.8% (*p* = 0.0019)
IMbrave150—atezolizumab + bevacizumab vs. sorafenib [76,80]	HCV 21% HBV 49% Non-viral 30% #	Geographical region (Asia excluding Japan vs. rest of the world) Macrovascular invasion or extrahepatic spread (presence vs. absence) Baseline AFP < 400 ng/mL vs. ≥400 ng/mL ECOG PS (0 vs. 1)	OS 19.2 vs. 13.4 (HR 0.66, 95% CI 0.52–0.85, *p* < 0.001) PFS 6.9 vs. 4.3 (HR 0.65, 95% CI 0.53–0.81, *p* < 0.001)	ORR 30% vs. 11% (*p* < 0.001) DoR 18.1 (95% CI 14.6-NE) vs. 14.9 (95% CI 4.9–17.0)
HIMALAYA—durvalumab vs. sorafenib and durvalumab + tremelimumab vs. sorafenib [77]	HBV 31% HCV 28% Nonviral 41%	Asia (excluding Japan) 39.7% and rest of world 60.3%. ECOG PS (0 vs. 1), AFP ≥ 400 (yes vs. no)), macrovascular invasion (yes vs. no), extrahepatic disease (yes vs. no), PD-L1 status pos. vs. neg.)	OS STRIDE 16.4 vs. sorafenib 13.8 (HR 0.78, 96% CI 0.65–0.92, *p* = 0.0035)	ORR STRIDE 20.1% vs. sorafenib 5.1% TTP 5.4 (95% CI, 3.8 to 5.6) in STRIDE arm, 3.8 (95% CI, 3.7 to 5.4) in durvalumab arm, and 5.6 (95% CI, 5.1 to 5.8) in Sorafenib arm
REFLECT—lenvatinib vs. sorafenib [72]	HCV 19% HBV 52.5% Alcohol 7.5% Other 7.9% Unknown 13%	Geographical region (Asia-Pacific or Western) ECOG PS (0 vs. 1) Presence or absence of macroscopic portal vein invasion and/or extrahepatic spread Body weight (<60 kg or ≥60 kg)	OS 13.6 vs. 12.3 (HR 0.92, 95% CI 0.79–1.06	PFS 7.4 vs. 3.7 (HR 0.66, 95% CI 0.57–0.77, *p* < 0.0001) TTP 8.9 vs. 3.7 (HR 0.63, 95% CI 0.53–0.73, *p* < 0.0001) ORR 24.1% vs. 9.2% (OR 3.13, 95% CI 2.15–4.56, *p* < 0.0001)
RESORCE—regorafenib vs. placebo [73]	HCV 21% HBV 38% Alcohol 24% Unknown 17% MASH 7% Other 7%	Geographical region (Asia vs. rest of world) Macrovascular invasion (yes vs. no) Extrahepatic spread (yes vs. no) Baseline AFP < 400 ng/mL vs. ≥400 ng/mL ECOG PS (0 vs. 1)	OS 10.6 vs. 7.8 (HR 0.68, 95% CI 0.50–0.79, *p* < 0.0001)	PFS 3.1 vs. 1.5 (HR 0.46, 95% CI 0.37–0.56, *p* < 0.0001) TTP 3.2 vs. 1.5 (HR 0.44, 95% CI 0.36–0.55, *p* < 0.0001) ORR 11% vs. 4% (*p* = 0.0047) DCR 65% vs. 36% (*p* < 0.0001)
CELESTIAL—cabozantinib vs. placebo [74]	HCV 24% HBV 38% HBV + HCV 2% Alcohol 24% MASH 9% Other 5% Unknown 16%	Etiology (HBV with or without HCV vs. HCV without HBV, or other) Geographical region (Asia or other) Extrahepatic spread and/or macrovascular invasion (yes vs. no)	OS 10.2 vs. 8 (HR 0.76; 95% CI 0.63–0.92, *p* = 0.005)	PFS 5.2 vs. 1.9 (HR 0.44, 95% CI 0.36–0.52, *p* < 0.001) ORR 4% vs. <1% (*p* = 0.009)
REACH-2—ramucirumab vs. placebo [75]	HCV 24% HBV 36% Alcohol 24% MASH 10% Cryptogenic 6% Other 9%	Geographical region (America, Europe, Australia, Israel vs. Asia, excluding Japan vs. Japan) Macrovascular invasion (yes vs. no) ECOG PS (0 vs. 1)	OS 8.5 vs. 7.3 (HR 0.710. 95% CI 0.53–0.95, *p* = 0.0199)	PFS 2.8 vs. 1.6 (HR 0.452, 95% CI 0.34–0.60, *p* < 0.0001) ORR 5% vs.1%, *p* = 0.1697) TTRP 3 vs. 1.6 (HR 0.427, 95% CI 0.31–0.58, *p* < 0.0001)

* Distribution is given for the treatment arm. # Non-viral included alcohol, other, unknown non-hepatitis B or C. SHARP: Sorafenib Hepatocellular Carcinoma Assessment Randomized Protocol, OS: overall survival, TTSP: time to symptomatic progression, TTRP: time to radiologic progression, DCR: disease control rate, HCV: hepatitis C virus, HBV: hepatitis B virus, ECOG: Eastern Cooperative Oncology Group Performance Score, PFS: progression-free survival, ORR: objective response rate, DoR: duration of response, NE: not evaluable, AFP: alpha fetoprotein, MASH non-alcoholic steatohepatitis.

## Data Availability

Not applicable.
